# Tuning electronic and magnetic properties through disorder in V_2_O_5_ nanoparticles

**DOI:** 10.1038/s41598-023-32642-0

**Published:** 2023-04-25

**Authors:** Sergio Correal, Daniel Hernández-Gómez, Andrea Steffania Esquivel, Alexander Cardona-Rodríguez, Andreas Reiber, Yenny Hernandez, Rafael González-Hernández, Juan Gabriel Ramírez

**Affiliations:** 1 Department of Physics, Universidad de los Andes, Bogotá, 111711 Colombia; 2 Escuela de Ingeniería, Ciencia y Tecnología, Universidad del Rosario, Bogotá, 111711 Colombia; 3 Department of Chemistry, Universidad de los Andes, Bogotá, 111711 Colombia; 4 Department of Physics, Universidad del Norte, Barranquilla, 081002 Colombia

**Keywords:** Electronic structure, Nanoparticles, Magnetic properties and materials

## Abstract

We report on the synthesis and characterization of V_2_O_5_ nanoparticles grown using a sol–gel method at different calcination temperatures. We observed a surprising reduction in the optical band gap from 2.20 to 1.18 eV with increasing calcination temperature from 400 to 500 °C. Raman and X-Ray diffraction measurements indicated slight changes in the lattice parameters induced by the growth process. However, density functional theory calculations of the Rietveld-refined and pristine structures revealed that the observed optical gap reduction could not be explained by structural changes alone. By introducing oxygen vacancies to the refined structures, we could reproduce the reduction of the band gap. Our calculations also showed that the inclusion of oxygen vacancies at the vanadyl position creates a spin-polarized interband state that reduces the electronic band gap and promotes a magnetic response due to unpaired electrons. This prediction was confirmed by our magnetometry measurements, which exhibited a ferromagnetic-like behavior. Our findings suggest that oxygen vacancies play a crucial role in band gap reduction and the promotion of a ferromagnetic-like response in an otherwise paramagnetic material. This provides a promising route to engineer novel devices.

## Introduction

Among vanadium oxides, vanadium pentoxide V_2_O_5_, which has the highest oxygen content^1^, is of paramount importance since its properties are highly sensitive to external factors, so it is used in photocatalysts^2^, chemical actuators^3^, optical fibers^4^, gas sensors^5–7^, and electrochromic devices^8^. The structure of V_2_O_5_ is best described as an arrangement of square pyramid VO_5_ units forming layers, which enables high ion storage capacity for batteries^9–12^. Recently, it has been found that reversible changes in V_2_O_5_ resistivity could be triggered by voltage^13^ or temperature^14^, which could be helpful for neuromorphic computing applications^15^ and photonic device architectures^16^. However, the driving mechanism for the changes in resistivity and in other properties is not yet well understood.

Vanadium pentoxide has been studied for several years experimentally. X-ray data indicate that V_2_O_5_ belongs to the orthorhombic structure with space group symmetry *Pmmn*^17^. Optical measurements suggest that the band gap of V_2_O_5_ thin film samples is around 2.3 eV and could be modified with the grain size or the thickness of the films^18–23^. These data rely on the Tauc plot method^24^ for the determination of the optical band gap. Magnetic measurements show that although V_2_O_5_ is diamagnetic in bulk^25^, it could be turned magnetic with the inclusion of sodium^26^ or oxygen vacancies^27^. However, little is known about the magnetic properties of V_2_O_5_ nanoparticles. The few available studies on magnetism in nanoparticles are about V_2_O_5_ with other compounds^28,29^, but there is, to the best of our knowledge, no one about pure V_2_O_5_ nanoparticles.

There are also various theoretical investigations about V_2_O_5_. Density Functional Theory (DFT) calculations show that the band structure of the material is characterized by a valence band that is mainly composed of oxygen 2*p* orbitals while the conduction band is made of vanadium 3*d* orbitals^30^. A distinctive feature of this material is the existence of a conduction band that is slightly separated from the others^30^. The electronic band gap obtained by DFT is around 1.8 eV^31^ while GW calculations give a value of around 4.0 eV^32–34^. Thus, compared to experimental evidence, there is still a controversy about the band gap energy of V_2_O_5_.

In this paper, we prepared V_2_O_5_ nanoparticles (NPs) using the sol–gel method^35^ at various calcination temperatures (T_CAL_). The T_CAL_ regulates the particle sizes and general properties of the NPs. We characterized the samples with X-ray diffraction, Raman and UV/vis spectroscopies, Scanning Transmission Electron Microscopy (STEM), and Vibrating Sample Magnetometry (VSM). We report a remarkable reduction of around 1 eV of the optical band gap of V_2_O_5_ NPs when increasing T_CAL_. By performing DFT calculations with Hubbard (U)^36^ and van der Waals (D3)^37,38^ corrections, we found that structural changes do not reduce the gap as much as observed experimentally. Instead, when including oxygen vacancies, the simulations show that a spin-polarized interband state appears, which decreases the band gap to values close to the experimental ones and induces a magnetic state. VSM measurements showed that the samples have a ferromagnetic-like behavior, which is probably attributed to oxygen vacancies and surface effects. Therefore, we show that the band gap and magnetism of V_2_O_5_ NPs can be tuned with T_CAL_ through oxygen vacancies, which might be relevant for future technological applications.

## Methods

### Experimental details

Vanadium pentoxide (V_2_O_5_) nanoparticles were synthesized using a non-aqueous sol–gel route^35^. A volume of 0.5 ml of vanadium oxytrichloride (VOCl_3_) of analytic degree was mixed with 20 ml of benzyl alcohol and stirred for 5 h at room temperature. The obtained blue solution was left to stand for 48 h and then heated at the target calcination temperature T_CAL_ for 12 h^35^. The T_CAL_ used were 400, 425, 438, 450, 475, and 500 °C for the first set of samples. Subsequently, with the second set of samples, the T_CAL_ used were 460, 500, and 540 °C. The first set of samples was used for the optical measurements while the second one was for magnetic measurements. The particle size change when increasing T_CAL_^39,40^.

X-ray diagrams of the first set were measured using a diffractometer PANalytical X’PERT PRO MPD with a Bragg Brentano configuration and an X-ray source Cu-Kα1 with 1.54060 Å wavelength. Raman spectra for the first set were obtained with a HORIBA XploRA One Raman spectrometer with shift values between 100 and 1200 cm^−1^. A laser light source of 638 nm was used in these measurements. The Raman spectrum peaks were analyzed utilizing Voigt distributions with Fityk software^41^. Microscopy analysis was performed using the TESCAN LYRA3 in a Scanning Transmission Electron Microscope (STEM) setup. Absorbance spectra were recorded with a spectrophotometer Analytik Jena Specord 50 Plus between 300 and 1100 nm. The sample powders were dissolved in isopropanol, obtaining an unsaturated solution^20,42^. Finally, magnetometry measurements were performed in powder samples in a vibrating sample magnetometer from LakeShore.

### Computational details

The calculations were based on density functional theory using the software Vienna Ab initio Simulation Package (VASP)^43–46^ adopting the exchange and correlation functional as parametrized by Perdew-Burke-Ernzerhof (PBE)^47^ under the Generalized Gradient Approximation (GGA). The Projector Augmented Wave (PAW) method^48^ was used to describe electron wave functions. An energy cutoff of 500 eV was sufficient for expanding the plane wave basis set. The orbital potentials method^36^ (DFT + U) was used to correct for the electron correlation in localized vanadium 3*d* orbitals, using *U* = 4 eV and *J* = 0 eV, as suggested by Scanlon et al*.*^31^ for V_2_O_5_. The van der Waals DFT + D3 method^37,38^ was also used to correct the weak interactions between the V_2_O_5_ layers. PyProcar^49^ and VASPKIT^50^ were used for the post-processing of VASP output data. The calculations were performed in bulk, so the characteristics of NPs, such as surface effects or interactions between them, were neglected (this approach has been successful with other systems^21,51^).

We first applied the computational method to the pristine α-V_2_O_5_ *Pmmn* structure with lattice parameters^17^ *a* = 3.564 Å, *b* = 11.512 Å, and *c* = 4.368 Å with a *k*-point sampling of 8 × 8 × 8. Using the experimental X-ray structures as inputs, we calculated the band structures and the absorption spectra under the independent particle approximation increasing the number of bands by four times the default value. Then, we simulated systems with three concentrations of oxygen vacancies: 1.1%, 2.5%, and 5.0%. These concentrations were achieved by removing one O1 oxygen from pristine supercells of sizes: 3 × 1 × 3 for 1.1%, 2 × 1 × 2 for 2.5%, and 2 × 1 × 1 for 5.0%. These systems were studied with spin-polarized calculations. The first Brillouin zone was sampled by 3 × 8 × 3, 4 × 8 × 4, and 4 × 8 × 8 k-point meshes for 1.1%, 2.5%, and 5.0% systems, respectively. Atomic positions were relaxed maintaining constant the experimental lattice parameters until the force on every ion was less than 0.01 eV Å^−1^. Oxygen vacancies in O2 and O3 positions were also investigated with the same methods as the O1 site.

## Results and discussions

**Figure 1:**
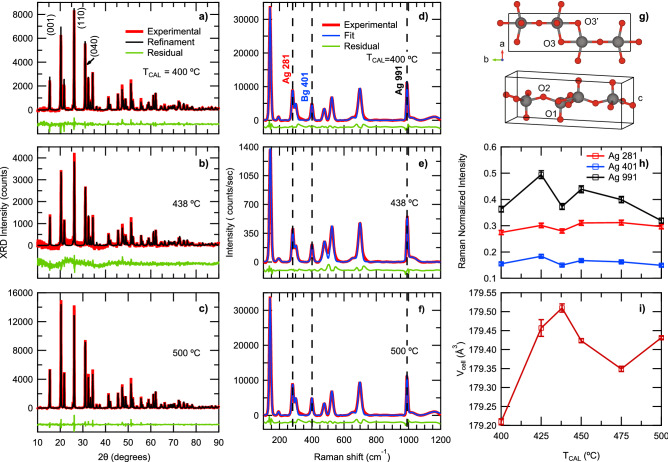
X-ray diffraction patterns of representative samples. T_CAL_ = (**a**) 400 °C, (**b**) 438 °C, and (**c**) 500 °C. All the X-ray diffraction peaks coincide with those reported for α-V_2_O_5_. Raman spectra for samples at T_CAL_ = (**d**) 400 °C, (**e**) 438 °C, and (**f**) 500 °C. The Raman peaks correspond to those ascribed to α-V_2_O_5_. The (**d**) panel indicates three Raman modes A_g_ or B_g_ with their positions in cm^-1^ that are associated with vanadyl oxygens O1. (**g**) The orthorhombic unit cell of V_2_O_5_ with vanadium atoms in grey and oxygen atoms in red. (**h**) Selected normalized Raman intensities as functions of T_CAL_. These normalized Raman intensities were calculated by dividing by the maximum intensity at 142 cm^−1^. The error bars were estimated by calculating the standard deviation from several measurements using the mode at 281 cm^−1^ for 450 °C as a reference. (**i**) Unit cell volume as a function of T_CAL_. A non-monotonic behavior around 438 °C is found in (**h**) and (**i**).

Figure 1a–c shows representative X-ray diffractograms of the samples. Using the Rietveld refinement, we identified a good overall match to a pure α-V_2_O_5_ phase^52–54^ with the most intense peaks of crystallographic planes (001), (110), and (040) indicated in Fig. 1a. Following the Scherrer method^55^, we estimated the primary particle sizes of the samples, which varied with T_CAL_ and are of the order of 60 nm (see Supplementary Table S1). STEM images confirmed the order of magnitude of the particle sizes (see Supplementary Figure S1). Figure 1d–f shows Raman spectra of the same samples. The Raman peaks from 100 to 1200 cm^−1^ fully coincide with those of the α-V_2_O_5_ phase^52,56^.

Figure 1i shows a non-monotonic behavior of the unit cell volume (*V*_*cell*_) as a function of T_CAL_. The *V*_*cell*_ increased from T_CAL_ 400 to 438 °C and decreased at 475 °C. The *V*_*cell*_ varied by a maximum of 0.17% of the pristine value of 179.2 Å^3^ reported in the literature^17^. Raman results also have a non-monotonic behavior after a Voigt peak fitting. Figure 1h shows the normalized Raman intensity for three representative peaks as a function of T_CAL_. Raman intensities have local minimums at 438 °C and the higher and smallest T_CAL_.

The non-monotonic behavior of the Raman intensities, and *V*_*cell*_ can be associated with structural changes induced by the synthesis process. Since the Raman intensities are proportional to the square of the polarizability derivative with respect to vibration coordinates^57^, it is expected that for larger bond distances, the Raman peaks become less intense. This can be noticed in the sample at T_CAL_ = 438 °C because it has the larger vanadyl distance so the weaker vanadyl Ag 991 cm^−1^ Raman mode (see Fig. 1d). In addition, the volume at 438 °C has a maximum because this sample has the largest *c*-lattice parameter. The Raman frequency of the vanadyl mode as a function of T_CAL_ also followed a similar trend as *V*_*cell*_, which indicates that the fabrication process produces slight structural changes, especially at 438 °C (see Supplementary Figure S2).

**Figure 2:**
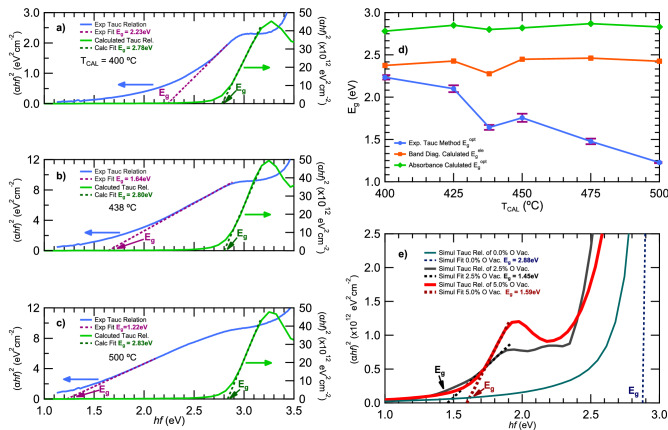
Optical properties obtained from the Tauc plot method and DFT calculations. (**a**–**c**) Comparison of Tauc plot relations obtained experimentally and plots calculated using DFT. (**d**) Experimental and ab initio optical band gap (from the Tauc plot method) and electronic band gap (calculated from DFT) as functions of T_CAL_. (**e**) Tauc plots simulated with 2.5% and 5.0% oxygen-deficient systems. Experimentally, the band gap reduces when increasing T_CAL_ and DFT calculations with the Rietveld-refined structures do not fully reproduce this trend. However, when including oxygen vacancies, the band gaps calculated are closer to the experimental values.

We investigated the correlations between the crystal structure and the optical properties of the V_2_O_5_ NPs by measurements of the optical absorption at room temperature. Figure 2a–c shows the absorption curves as blue lines. The optical band gap *E*_*g*_^*opt*^ was determined using the Tauc plot method^24^ to these curves assuming an indirect transition. The results are summarized in Fig. 2d. Notably, the optical band gap decreases considerably when increasing T_CAL_. The total change between the T_CAL_ = 400 and 500 °C is close to 1 eV. These values appear to be extremely small compared to values reported by V_2_O_5_ in bulk using the same Tauc plot technique^58^. Additionally, at T_CAL_ = 438 °C, there is a minimum of *E*_*g*_^*opt*^. This minimum corresponds to the sample with the greater vanadyl distance and unit cell volume (see Fig. 1). This may suggest that local structural changes may control the optical properties. However, it appears that the overall decrease of the optical band gap cannot be solely explained by the structural parameters obtained from the X-ray and Raman measurements. To identify the contributions to the optical band gap due to the lattice structure, we carried out first-principles calculations.

### Computational results

**Figure 3:**
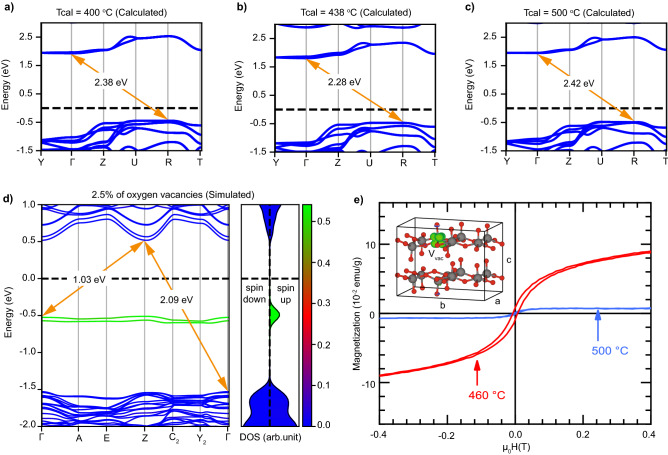
Electronic structures and magnetic measurements. (**a**–**c**) Representative DFT band structures calculated from Rietveld-refined structures at different T_CAL_. (**d**) Simulated band structure and density of states of the 2.5% oxygen-deficient system, projected onto V_vac_ *d* orbitals. The color bar indicates the contributions of the *d* orbitals. When oxygen vacancies are considered, a spin-polarized interband state appears, which reduces the band gap. (**e**) VSM measurements at T_CAL_ = 460 and 500 °C, which indicate that the NPs have a ferromagnetic-like behavior. The inset in (**e**) corresponds to the relaxed crystal structure of the 2.5% oxygen-deficient system and its spin density as a green surface. This spin density was localized at the vanadium atom which lost its vanadyl O1 oxygen.

We first calculated the band structure of the V_2_O_5_ pristine crystal (see Supplementary Figure S3), which shows that vanadium 3*d* orbitals (especially *d*_*xy*_) predominate the conduction band and that there is one *d*-band slightly separated from the others. We found an indirect electronic band gap of *E*_*g*_^*ele*^ = 2.42 eV, which is in good agreement with experimental^23^ (2.33 eV) and theoretical data^59^. Then, we used the refined X-ray crystal structures at different T_CAL_ as inputs and calculated their band structures (see Fig. 3a–c). Overall, the band structures show a similar behavior compared to the pristine one. From the band structures, we calculated the corresponding *E*_*g*_^*ele*^ values. Figure 2d shows how *E*_*g*_^*ele*^ compares with the corresponding experimental values (see the orange squared symbols). The *E*_*g*_^*ele*^ values versus T_CAL_ match moderately with the experimental results at T_CAL_ = 400 °C. Furthermore, the calculations appear to capture the minimum *E*_*g*_^*ele*^ at 438 °C.

However, the overall values calculated for the band gap are much higher than the ones observed experimentally. One reason may be due to the presence of excitons which may change the optical band gap considerably^33^. We then proceeded to calculate the optical band gap from the calculations. We calculated the optical absorption curve under the independent particle approximation (see green curves in Fig. 2a–c) and employed the Tauc plot method to estimate the optical band gap, as in the experimental case. Figure 2d show the calculated band gap versus T_CAL_ (green diamond symbol). All values are close to 2.8 eV with little change. Furthermore, they are far away from the ones obtained experimentally. Therefore, the structural changes observed in X-ray diffraction are minor and do not fully explain the behavior of the band gap with T_CAL_. Similarly, Kang et al*.*^60^ found that augmenting the temperature of α-V_2_O_5_ film samples while measuring, largely modifies their optical properties, but structural modifications do not completely explain these changes.

A reason that might explain the differences between theory and experiment is the existence of oxygen vacancies^13,14,61^. Exposing vanadium pentoxide samples to 1 min of air creates oxygen vacancies, which changes the material properties^62^. Thus, it is unlikely that the various synthetic methods to prepare this material, including the sol–gel synthesis^35^, produce V_2_O_5_ with total purity but instead with a certain degree of oxygen vacancies. Accordingly, the experimental properties available in the literature, such as the band gap, might be affected by this situation. Therefore, we simulated three V_2_O_5_ systems with 1.1%, 2.5%, and 5.0% concentrations of oxygen vacancies. The energy required to remove an O1 oxygen required about 2 eV less than the O2 or O3 types (see Supplementary Table S2). This result is consistent with previous reports^31^. Thus, we focused our calculations on the O1 defect.

After relaxation, the new geometries of the 1.1% and 2.5% systems formed a new bond between the vanadium vacancy site (V_vac_) and the O1 of an adjacent layer (see inset of Fig. 3e and Supplementary Figure S4). These results are similar to the ones of Scanlon et al*.*^31^, who also found a new bond between layers. The 5.0% system had slight local changes since in this case there is no adjacent O1 oxygen (see Supplementary Table S3 for specific distances and angle variations). These geometrical changes might be explained by the chemical bonds formed. The V-O1 bond is analogous to the vanadyl group VO^2+^, which appears in many inorganic species of this metal. This group is commonly encountered with four other ligands, forming a square pyramid geometry such as the [VOCl_4_]^2-^ anion^63^. The vanadium coordination number in these molecules is five. When an O1 atom is removed in V_2_O_5_, the atom V_vac_ (which acts as a Lewis acid) becomes four-coordinated, forming the less stable square geometry. Therefore, V_vac_ binds to O1_layer2_ and recovers the coordination number of five and the square pyramid structure (recall atom labels from Supplementary Figure S4).

The relaxed oxygen-deficient structures also showed a change in their symmetry operations. The symmetry of these systems shifted from pristine^22^ *Pmmn* ${D}_{2h}^{13}$ to *Pm* ${C}_{s}^{1}$, as calculated using FINDSYM^64^. We also simulated the X-Ray diffractograms of these systems using the VESTA^65^ software and found no difference against the pristine structure (see Supplementary Figure S5). Thus, we confirmed that X-Ray diffraction cannot detect the studied vacancy concentrations.

When including oxygen vacancies, another relevant aspect to consider are electric charges. The process of releasing one oxygen from V_2_O_5_ creates a vacancy site and lefts 2 electrons in excess in the lattice. According to the spin densities in the inset of Fig. 3e (and Supplementary Figure S4), the two released electrons localize mainly on V_vac_. Thus, one might assign to V_vac_ an oxidation state of + 3 for 1.1% and 2.5% systems or + 4 for the 5.0% system. Such inferences correspond to the fact that signals of V^4+^ and V^3+^ are observed in X-ray photoelectron spectra when heating V_2_O_5_ thin films under an ultrahigh vacuum chamber^66^. The results also align with other calculations^67^. In addition, *V*_*cell*_ tends to expand with oxygen vacancies because reduced cations have larger ionic radii^68^, and *V*_*cell*_ was indeed greater than the pristine value of 179.2 Å^3^ for various T_CAL_ (see Fig. 1i).

Figure 2e shows the simulated Tauc plot relation for the 2.5% oxygen-deficient system, and Fig. 3d presents its electronic band structure. The band structure shows an interband state which reduces ~ 1 eV the band gap compared to the pristine value. Similarly, the simulated Tauc plot curve of this system has a straight segment at low energies associated with a band gap energy of 1.45 eV. Thus, both the electronic and the Tauc-simulated band gap give values that are much closer to the experimental ones at high T_CAL_. These findings are also in agreement with X-ray and ultraviolet photoelectron spectroscopies signals of an interband state at 1.3 eV above the valence band of thin film V_2_O_5_ samples^66,69^.

The interband state is also below Fermi energy and is populated by excess electrons. This suggests that V_2_O_5_ acts as an n-type semiconductor, which agrees with the literature^5,70^. Figure 3d and Supplementary Figure S6 present partial densities of states of the oxygen-deficient systems and reveal that the interband state is mainly composed of V_vac_ 3*d* orbitals, specifically *d*_*yz*_ and *d*_*xz*_ ones for the 1.1% and 2.5% systems. For the 5.0% system, two interband states resulted. The band farther from the valence is mainly made by the *d*_*xz*_ orbital, and the one closest to the valence is made by the ${d}_{{z}^{2}}$ orbital. Similar results were obtained by Blum et al*.*^14^ and Laubach et al*.*^69^. These results support the hypothesis that mainly oxygen vacancies and, to a less extent, structural changes are the origin of the reduction of the band gap with T_CAL_.

Moreover, the calculations on the oxygen-deficient systems show that their interband state is spin-polarized, so they have a magnetic behavior. To verify this, we performed VSM measurements of samples at T_CAL_ = 460 and 500 °C since the optical band gap showed greater changes at high temperatures. Figure 3e shows the magnetization as a function of the external magnetic field. Indeed, the results indicate that V_2_O_5_ NPs have a ferromagnetic-like behavior. The saturation magnetization was considerably greater at 460 °C than at 500 °C. This implies that T_CAL_ tunes the magnetic properties of V_2_O_5_ NPs. However, further studies should be performed to fully understand how the magnetization scales with T_CAL_, including a complete range of temperatures.

It is known that many nonmagnetic metal oxides exhibit ferromagnetic ordering when they form NPs because of the interactions between spin moments resulting from oxygen vacancies at their surfaces^71^. Therefore, the V_2_O_5_ ferromagnetic-like state observed experimentally might be ascribed to oxygen vacancies on the surfaces of the NPs. Our magnetic results also align with other experimental evidence such as the ferromagnetic response found in oxygen-deficient thin film V_2_O_5_ systems^27^. The results are also supported by other theoretical calculations that indicate that the ferromagnetic solution is more stable than the nonmagnetic or antiferromagnetic ones in oxygen-deficient V_2_O_5_ systems^25^.

The magnetic results were understood based on the O1 type of oxygen vacancies, but O2 and O3 defects may also exist. The O1 vacancy requires the least energy and is the most probable to be present^25,31^ (see Supplementary Table S2). Nevertheless, we also studied the electronic structure of the O2 and O3 systems for 5.0% and 2.5% concentrations. Intriguingly, the calculations show that oxygen vacancies at these positions do not produce a spin imbalance and thus do not contribute to the material's magnetic properties (see Supplementary Figure S8). This is attributed to the distinct chemical environment of the O1 vacancy compared to the O2 and O3 ones. In the O1 vacancy, the excess charge is localized mainly on one vicinal vanadium atom. However, with the O2 and O3 types, the electrons in excess are delocalized mainly on the two or three vicinal vanadium atoms. Therefore, the magnetic properties observed experimentally are only ascribed to O1 vacancies.

On the other hand, the saturation magnetization might be used to approximate the concentration of oxygen vacancies. Because each oxygen vacancy adds 2 µ_B_ to the system, there is an exact linear relationship between the magnetization per molecular formula (*M*) and the oxygen vacancy percentage (*C*), which is *M* = *αC*, with *α* = 0.1 µ_B_, between the concentrations studied. This fact was also previously reported in the literature^25^. Assuming that the NPs are spherical and have a size equal to the average primary particle sizes of Supplementary Table S1, we can roughly estimate the *C* on the surfaces of the NPs using their experimental magnetization (see Supplementary Note for more detail). Following this reasoning, we estimate concentration values of 0.25% and 0.02% for T_CAL_ = 460 and 500 °C, respectively. These values appear to agree with the percentual changes in *V*_*cell*_. As mentioned previously, oxygen vacancies are expected to increase *V*_*cell*_, and the cell volume of the samples at T_CAL_ = 450 and 500 °C expanded by 0.12% (see Fig. 1i). This is the same order of magnitude of the concentration estimated with the magnetization at T_CAL_ = 460 °C.

With these results, a question arises about how the concentration of oxygen vacancies relates to T_CAL_. Intuition suggests that a greater T_CAL_ can increase *C* since a higher T_CAL_ provides more thermal energy that can promote reduction–oxidation reactions, but these reactions may also result in the recombination of vacancies due to thermal energy. If we assume a direct relationship between *C* and *V*_*cell*_, Fig. 1i indicates that *C* varies non-monotonically with T_CAL_. This suggests that at higher temperatures, the fabrication process may not promote as many vacancies as at intermediate temperatures. In addition, the *C* value estimated with the magnetization reduced from 0.25% at 460 to 0.02% at 500 °C. Thus, the dependence of oxygen vacancy concentration with T_CAL_ is probably not straightforward, and more studies are required to address this correlation.

## Conclusions

We have synthesized V_2_O_5_ nanoparticles using a non-aqueous sol–gel method at different calcination temperatures and characterized their properties using structural, optical, and magnetic techniques. Our results demonstrate that the calcination temperature plays a crucial role in determining the properties of the nanoparticles. The nanoparticles' optical band gap decreased from 2.20 to 1.18 eV with an increase in calcination temperature from 400 to 500 °C. While the structural changes induced by calcination were found to be minor, our DFT + U + D3 calculations suggest that the reduction in the band gap is mainly due to the presence of oxygen vacancies at the vanadyl position that induce a spin-polarized interband state, resulting in the promotion of unpaired electrons. Furthermore, our magnetometry measurements reveal that the nanoparticles exhibit a ferromagnetic-like behavior, which may be attributed to oxygen vacancies. However, further studies are needed to understand the details of the magnetism observed and to quantify the number of vacancies induced by the growth process. Our study highlights the importance of oxygen vacancies in modifying the band gap and magnetic properties of V_2_O_5_ nanoparticles and suggests that these properties could be harnessed for developing novel devices for photonics and neuromorphic computing.

## Supplementary Information

Supplementary Information.

## Data Availability

The datasets generated and analyzed during the current study are available in the Crystallography Open Database repository, entries: 3000425, 3000426, 3000427, 3000428, 3000429, and 3000430, for calcination temperatures 400, 425, 438, 450, 475, and 500 °C, respectively.
