# Supplementary material for: Tuning electronic and magnetic properties through disorder in V2O5 nanoparticles
Source: Sci Rep. 2023 Apr 25;13:6752. doi: 10.1038/s41598-023-32642-0 (PMC10130179; doi:10.1038/s41598-023-32642-0)
Supplement: Supplementary file 1 — Supplementary Information. [file 41598_2023_32642_MOESM1_ESM.pdf]

Tunning electronic and magnetic properties through disorder in V_2O_5 nanoparticles

Sergio Correal[1,+], Daniel Hernández-Gómez[1,+], Andrea Steffania Esquivel[1,+], Alexander Cardona-Rodríguez[2], Andreas Reiber[3], Yenny Hernandez[1], Rafael González-Hernández[4], and Juan Gabriel Ramírez[1,*]

[1]Department of Physics, Universidad de los Andes, Bogotá, 111711, Colombia
[2]Escuela de Ingeniería, Ciencia y Tecnología, Universidad del Rosario, Bogotá, 111711, Colombia
[3]Department of Chemistry, Universidad de los Andes, Bogotá, 111711, Colombia
[4]Department of Physics, Universidad del Norte, Barranquilla, 081002, Colombia

[*]jgramirez@uniandes.edu.co
[+]these authors contributed equally to this work.

Supplementary Information

T_{CAL} (°C)	Primary particle size (nm)
400	57
425	57
438	50
450	59
475	67
500	64

Supplementary Table S1. Primary particle sizes. The sizes were estimated by the crystallite sizes according to the Scherrer method taking the value of the Scherrer constant as k = 0.9.

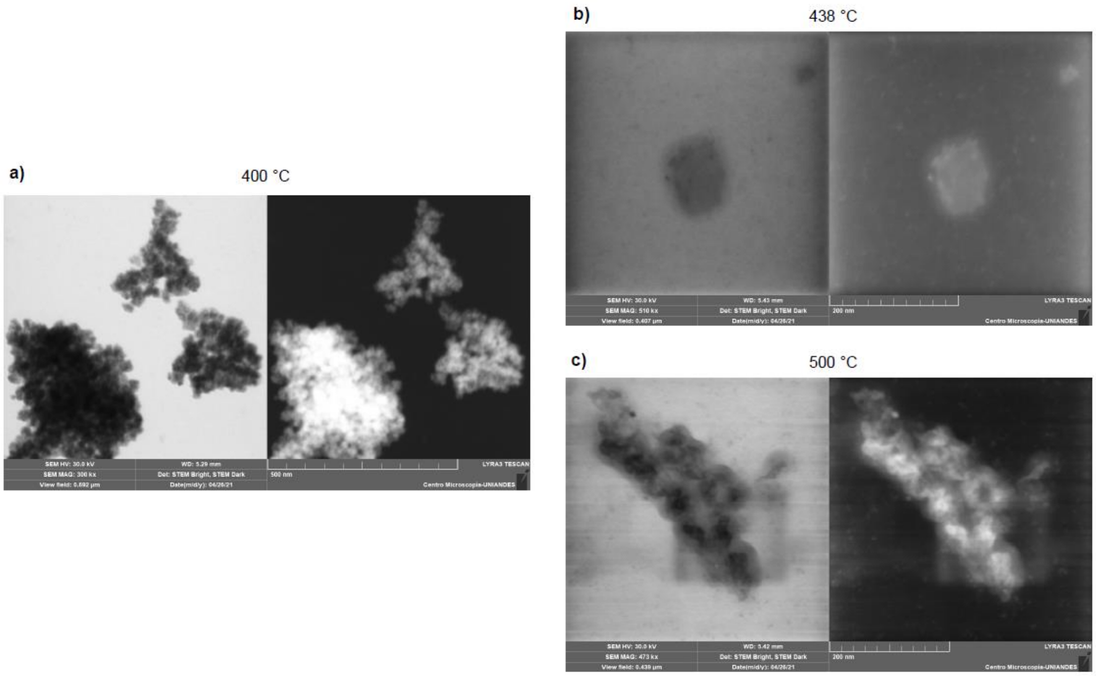

Supplementary Figure S1. STEM images. (a) T_{CAL} = 400 °C, (b) 438 °C, and (c) 500 °C.

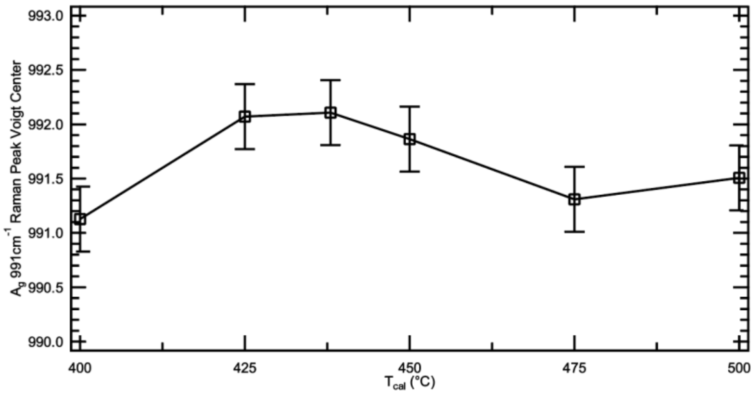

Supplementary Figure S2. Raman frequency of the vanadyl mode as a function of T_{CAL}. The error bars were estimated using a Voigt fitting procedure. The frequency followed a similar trend as V_{cell}.

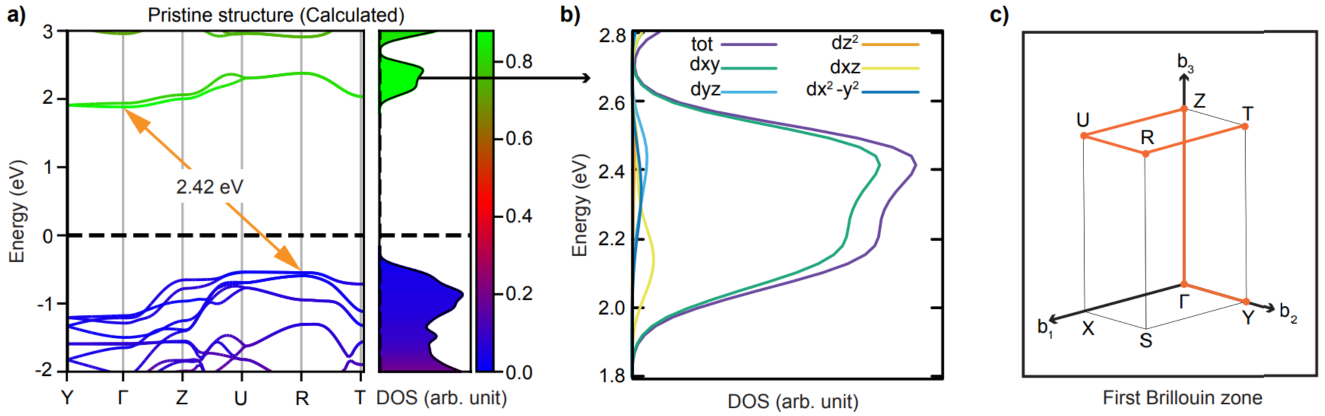

Supplementary Figure S3. Electronic structure of pristine V_2O_5. (a) Band structure and density of states projected onto *d* orbitals of vanadium atoms. The color bar indicates the contributions of these orbitals. (b) Partial density of states showing the contributions of vanadium *d* orbitals. The d_{xy} orbital shows the greatest contribution to the interband state. (c) First Brillouin zone which has the selected k path highlighted in orange.

Type of oxygen vacancy	2.5 % system Formation energy (eV)	5.0 % system Formation energy (eV)
O1	1.12	2.22
O2	4.12	4.28
O3	3.65	3.84

Supplementary Table S2. Formation energies of oxygen vacancy types. The values are calculated according to the reaction $V_V^x + O_O^x \rightarrow V_V^x + 0.5\ O_{2\ (g)} + v_O^{\cdot\cdot} + 2e'$ where *v* refers to the vacancy in Kröger–Vink notation. The O1 vacancy requires the least energy.

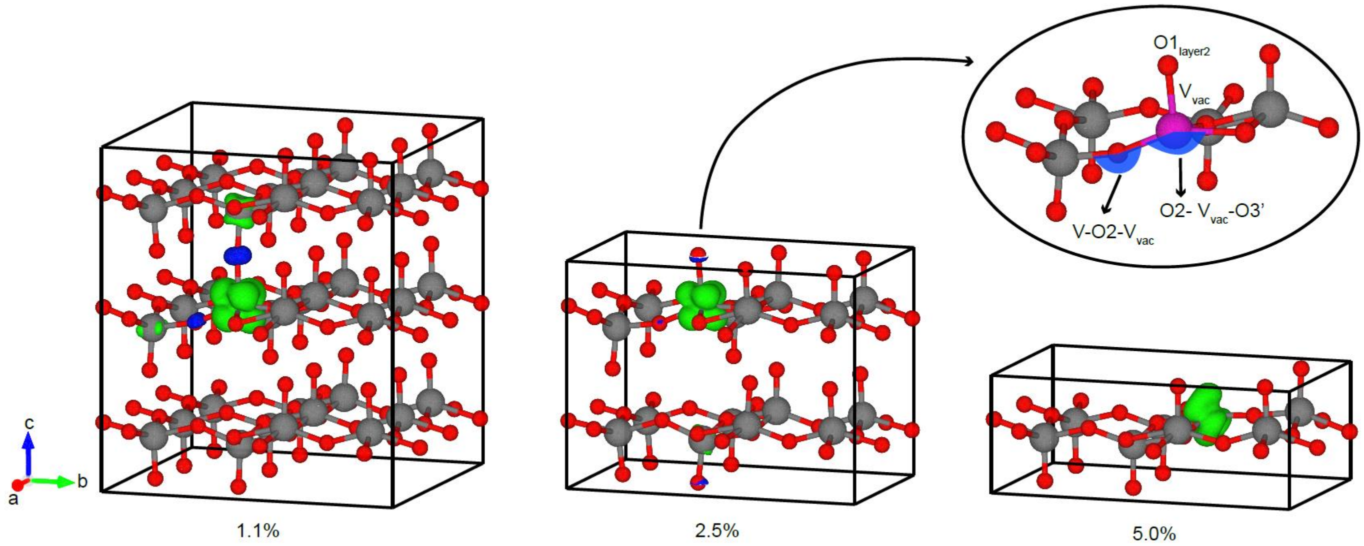

Supplementary Figure S4. Relaxed crystal structures and spin densities of oxygen-deficient systems. Gray atoms correspond to vanadium and red ones to oxygen. The spin density, which is the difference between spin up and down densities, is shown as colored surfaces. Green surfaces correspond to regions where there is a high probability to find an electron with spin up and blue surfaces to spin down. Green surfaces suggest that electrons in excess are localized on V_{vac} atom. The inset corresponds to an enlarged picture of the vicinity of V_{vac} for the 2.5% system with important labels (only some atoms are shown).

System	$V_{vac} - O1$ (Å)	$V - O2 - V_{vac}$ (deg)	$O2 - V_{vac} - O3'$ (deg)
Pristine	2.793	148.9	150.5
1.1%	1.946 (-30%)[a]	193.0 (+30%)	204.5 (+36%)
2.5%	1.954 (-30%)	196.4 (+32%)	203.0 (+35%)
5.0%	NA[b]	175.0 (+18%)	171.1 (+14%)

Supplementary Table S3. Representative changes of distances and angles of oxygen-deficient systems. The labels are specified in the inset of Supplementary Figure S4. [a]The percentage in parenthesis corresponds to the difference between the relaxed value and the pristine reference. [b]For the 5.0% oxygen deficient system, the distance $V_{vac} - O1$ does not apply because in this case there is not an adjacent O1 to V_{vac}.

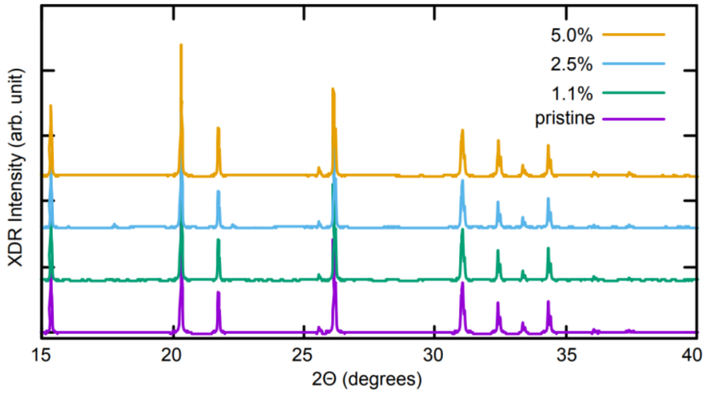

Supplementary Figure S5. Simulated X-ray diffractograms for oxygen-deficient systems performed in VESTA. The diffractograms of the three simulated oxygen vacancy concentrations show no significant differences against the pristine one.

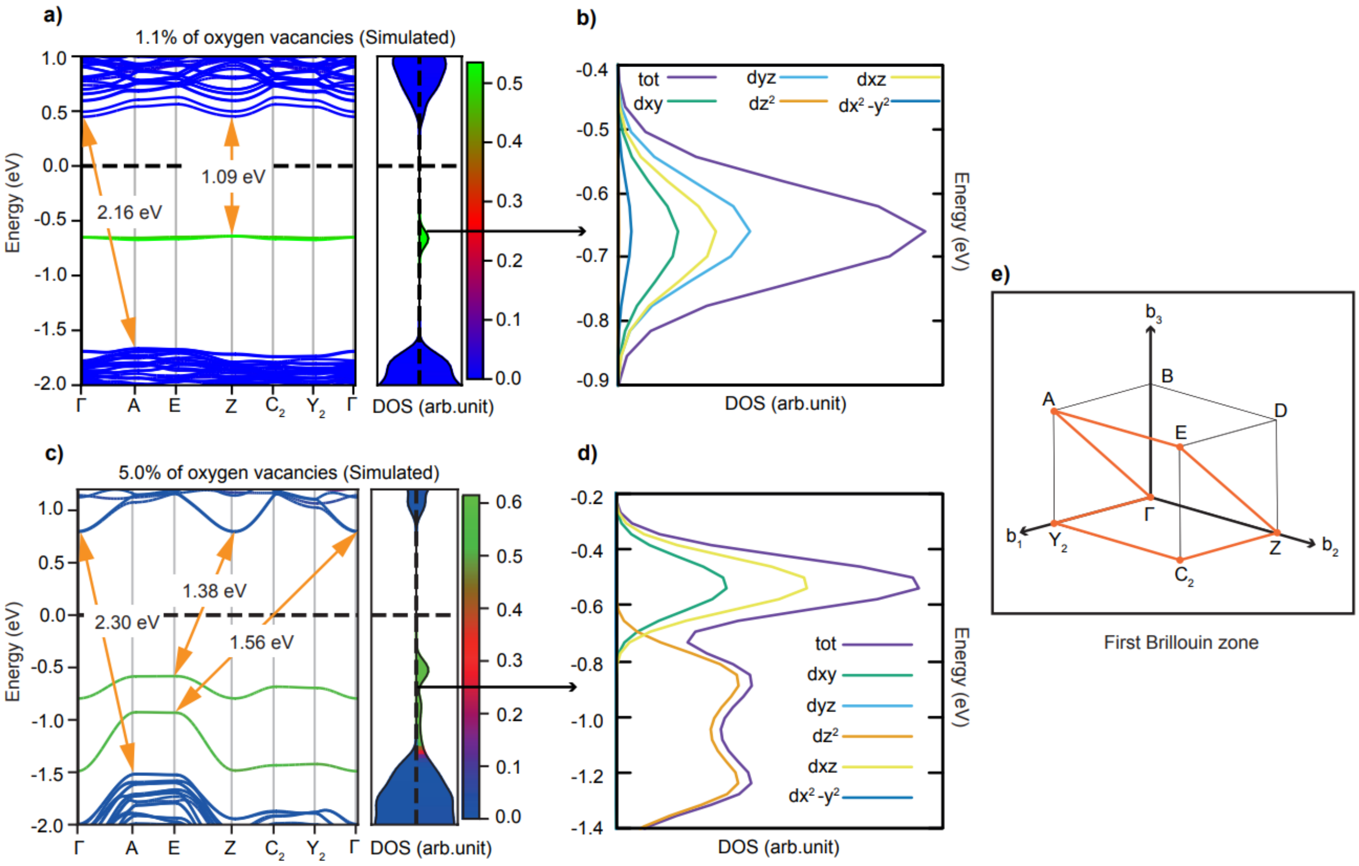

Supplementary Figure S6. Electronic structures of the 1.1 % and 5.0 % oxygen-deficient systems. Band structure together with density of states, and partial density of states projected onto *d* orbitals of vanadium atom with the O1 oxygen vacancy for the 1.1% (a-b) and 5.0% (c-d) oxygen deficient systems. The color bar indicates the contributions of these orbitals. (e) First Brillouin zone with the selected k path highlighted in orange.

Supplementary Note. Calculation of oxygen vacancy concentrations with the experimental magnetization.

Let ϕ be the total magnetic moment, m the mass, and N the total number of nanoparticles of the sample. Suppose all the nanoparticles are spherical with diameter d and the oxygen vacancies are only on their surfaces. We approximate the volume of a thin film of the nanoparticle's surface as the image below.

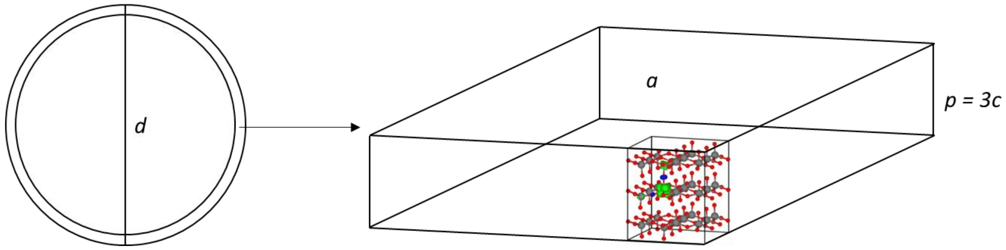

Supplementary Figure S7. Sketch of a V_2O_5 nanoparticle and a film of its surface.

The film has a face with an area a that corresponds to the surface area of the nanoparticle $a = \pi d^2$. The thickness of the film p is taken as three times the lattice parameter c. Let n be the number of molecular formulas of V_2O_5 in the superficial film of one nanoparticle and $M' = \phi/m$ the experimental magnetic saturation of the samples. The concentration of oxygen vacancies in the system is

$$C = \frac{number\ of\ oxygen\ vacancies}{number\ of\ oxygens\ without\ vacancies} * 100\%$$

In this context, we wish to determine C given M'. According to the DFT calculations, there is an exact linear relationship between the magnetization per molecular formula $M = \phi/Nn$ and the concentration of oxygen vacancies: $M = \alpha C$, where $\alpha = 0.1\ \mu_B$. The first task we must do is to find n, the number of molecular formulas in the film. To do this, let V be the volume of the film and v the volume of a unitary cell. Because there are 2 molecular formulas per unitary cell of V_2O_5, we have that $n = 2V/v = 2ap/v = 2\pi d^2\ p/v$. Using the linear relation, we have that

$$C = \frac{\phi}{nN\alpha} = \frac{\phi v}{2\pi d^2 pN\alpha} = \frac{mM'v}{2\pi d^2 pN\alpha}$$

Now, we must find N. To do this, let ρ be the mass density of vanadium pentoxide. The volume of one nanoparticle is $\pi d^3/6$, so its mass is $\rho\pi d^3/6$. Therefore, the number of nanoparticles in a sample of mass m is $N = 6m/\rho\pi d^3$. Finally, the concentration of oxygen vacancies turns out to be

$$C = \frac{mM'v}{2\pi d^2 pN\alpha} = \frac{\rho\pi d^3 mM'v}{12\pi d^2 pm\alpha}$$

which can be simplified to

$$C = \frac{\rho d M'v}{12p\alpha}$$

For the calculations, we used the mass density of V_2O_5 reported in the literature ρ = 3.357 g/cm^3, the average of the particle sizes d = 60 nm of Supplementary Table S1, and the lattice parameters a = 3.564 Å, b = 11.512 Å, and c = 4.368 Å, for $v = abc$ and $p = 3c$.

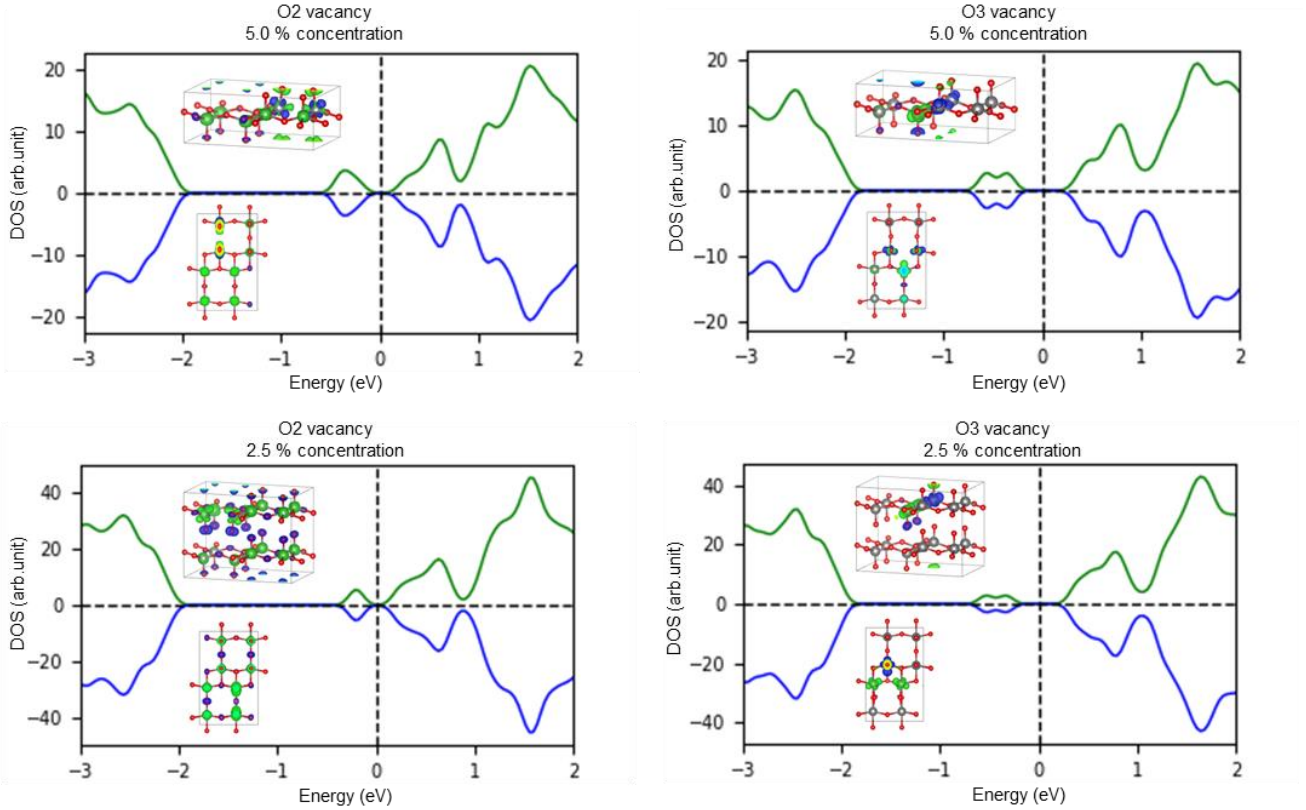

Supplementary Figure S8. Densities of states of O2 and O3 vacancies. The insets correspond to the relaxed crystal structures and the spin densities as surfaces, which are colored green for spin up and blue for spin down. Contrary to the O1 type, the O2 and O3 vacancies do not show a spin imbalance.

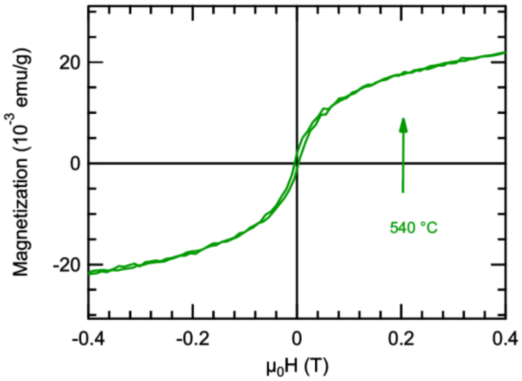

Supplementary Figure S9. VSM measurement of a sample at T_{CAL} = 540 °C.
